# Influence of denaturants on amyloid β42 aggregation kinetics

**DOI:** 10.3389/fnins.2022.943355

**Published:** 2022-09-20

**Authors:** Tanja Weiffert, Georg Meisl, Samo Curk, Risto Cukalevski, Anđela Šarić, Tuomas P. J. Knowles, Sara Linse

**Affiliations:** ^1^Department of Biochemistry and Structural Biology, Lund University, Lund, Sweden; ^2^Yusuf Hamied Department of Chemistry, Centre for Misfolding Diseases, University of Cambridge, Cambridge, United Kingdom; ^3^Yusuf Hamied Department of Chemistry, University of Cambridge, Cambridge, United Kingdom; ^4^Department of Physics and Astronomy, University College London, London, United Kingdom; ^5^Cavendish Laboratory, Department of Physics, University of Cambridge, Cambridge, United Kingdom

**Keywords:** misfolding, aggregation mechanism, denaturant, urea, guanidinium hydrochloride, self-assembly

## Abstract

Amyloid formation is linked to devastating neurodegenerative diseases, motivating detailed studies of the mechanisms of amyloid formation. For Aβ, the peptide associated with Alzheimer’s disease, the mechanism and rate of aggregation have been established for a range of variants and conditions *in vitro* and in bodily fluids. A key outstanding question is how the relative stabilities of monomers, fibrils and intermediates affect each step in the fibril formation process. By monitoring the kinetics of aggregation of Aβ42, in the presence of urea or guanidinium hydrochloride (GuHCl), we here determine the rates of the underlying microscopic steps and establish the importance of changes in relative stability induced by the presence of denaturant for each individual step. Denaturants shift the equilibrium towards the unfolded state of each species. We find that a non-ionic denaturant, urea, reduces the overall aggregation rate, and that the effect on nucleation is stronger than the effect on elongation. Urea reduces the rate of secondary nucleation by decreasing the coverage of fibril surfaces and the rate of nucleus formation. It also reduces the rate of primary nucleation, increasing its reaction order. The ionic denaturant, GuHCl, accelerates the aggregation at low denaturant concentrations and decelerates the aggregation at high denaturant concentrations. Below approximately 0.25 M GuHCl, the screening of repulsive electrostatic interactions between peptides by the charged denaturant dominates, leading to an increased aggregation rate. At higher GuHCl concentrations, the electrostatic repulsion is completely screened, and the denaturing effect dominates. The results illustrate how the differential effects of denaturants on stability of monomer, oligomer and fibril translate to differential effects on microscopic steps, with the rate of nucleation being most strongly reduced.

## Introduction

The folding, assembly and stability of the different states of a protein are of fundamental importance in protein chemistry. Many natural proteins are only marginally stable and may be present at or close to supersaturated concentrations *in vivo*, which makes them susceptible to aggregation and amyloid formation. Amyloid formation is associated with a large number of common human disorders, including Alzheimer’s disease, Parkinson’s disease, type II diabetes, and several systemic amyloidosis. The aggregation propensity and subsequently the diseases caused by amyloid formation are determined by the stability of the native proteins or peptides relative to the stability of the fibrils (Ramirez-Alvarado et al., 2000). While the folding reaction is unimolecular, aggregation is a multi-molecular reaction for which the total protein or peptide concentration comes in as a critical determinant. It is therefore key to identify and understand the role of stabilising as well as destabilising factors in the delicate balance that governs folding versus misfolding and aggregation. Protein aggregation is governed by the same intermolecular forces that govern protein folding and stability, e.g., hydrogen bonds, van der Waals interactions, electrostatic interactions, changes in configurational entropy and the hydrophobic effect (Makin et al., 2005; Williams et al., 2006; Knowles et al., 2007; Colvin et al., 2016). The propensity of a polypeptide chain to form amyloid fibrils is therefore dependent on the amino acid sequence, the protein concentration, the composition of the surrounding solution and physico-chemical parameters like temperature, pH, etc. (Betts et al., 2008; Shammas et al., 2011; Marek et al., 2012; Meisl et al., 2014, 2016a, 2017a; Abelein et al., 2016) and the presence of surfaces (Schladitz et al., 1999; Linse et al., 2007; Vácha et al., 2014). The aggregation process and the rates of the underlying steps are greatly influenced by the solution conditions and fibril formation under different solution conditions has been studied extensively, e.g., to explore the effect of ionic strength (Meisl et al., 2017a), pH (Meisl et al., 2016a), and temperature (Cohen et al., 2018).

The addition of denaturants to an aggregation-prone protein may affect the aggregation process, its rate as well as the stability of the formed fibrils (Narimoto et al., 2004; Baldwin et al., 2011; Vettore and Buell, 2019; Buell, 2022; Sønderby et al., 2022). Fibril formation is a multi-molecular self-assembly process involving coupled folding and assembly. Denaturants may modify equilibrium properties, e.g., may cause an increase in protein solubility, but can also modify the kinetics by affecting the stability of transition states relative to the reactants; the same ideas as in phi value analysis of folding kinetics may also apply here (Dagget and Fersht, 2000). Despite the long-established use of denaturants in studies of protein stability and folding, the mechanistic understanding of how these denaturants affect, or interact, with proteins remains elusive. The general belief is that urea and guanidinium hydrochloride (GuHCl) are preferentially accumulated in the vicinity of the polypeptide surface, resulting in higher denaturant concentrations near the polypeptide surface than in the bulk (Schellman, 1987; Shimizu and Chan, 2002; Bennion and Daggett, 2003; Modig et al., 2003). Unfolded peptides have larger preferential interaction with denaturants than the folded structure hence the denaturants may destabilise amyloid fibrils by lowering the free energy of the unfolded monomeric state with respect to the folded and assembled fibrillar state. While the two denaturants are structurally similar, GuHCl is charged while urea is not, thus distinct effects are expected for their interaction with charged proteins, such as Aβ.

The overall effect of denaturants on the aggregation rate depends, above all, on the conformation of the native protein, and ranges from acceleration (Hamada and Dobson, 2002) to retardation (Khan et al., 2017) of aggregation. For example, the addition of urea to Cu/Zn superoxide dismutase (SOD1) increased the lag time (i.e., decreased the overall rate) for fibril formation indicating that, in this case, the increased population of unfolded species caused by the addition of urea is not the dominant factor determining the aggregation onset (Khan et al., 2017). By contrast, the aggregation rate of β-lactoglobulin was found to increase as the urea concentration was increased up to 5 M. Below 5 M urea, the population of β-lactoglobulin was shifted from the native to toward the unfolded state resulting in accelerated aggregation, while above 5 M urea the aggregation rate decreases since urea can solvate the unfolded state to a sufficient degree to render the aggregated state kinetically or thermodynamically unfavorable (Hamada and Dobson, 2002). Similarly, it has been shown that the aggregation rate of hen egg-white lysozyme increases by addition of up to 3 M GuHCl (Vernaglia et al., 2004); at GuHCl concentrations above 3 M the aggregation rate decreases. A striking example of a protein for which unfolding promotes aggregation is transthyretin (TTR), the aggregation of which is linked to familial amyloid polyneuropathy (FAP). Natively folded TTR is a tetramer, and the dissociation into monomers and partial unfolding accelerates amyloid formation. The disease can be influenced by adding molecules stabilising the native TTR tetramer. One such small molecule, tafamidis, slows down the progression of peripheral and autonomic neuropathy for patients suffering from FAP and has recently been approved for clinical use (Coelho et al., 2016). This example illustrates how a mechanistic understanding of the aggregation process can guide therapeutic intervention.

The microscopic steps involved in the aggregation process starting from unfolded monomeric amyloid β42 (Aβ42) peptide, involved in Alzheimer’s disease, have been studied thoroughly (Cohen et al., 2013) and a combined theoretical and experimental approach to study intrinsic and extrinsic effects on the aggregation process is well established (Meisl et al., 2016a,b). *In vitro*, Aβ42 fibrils are formed through a nucleated growth process where new fibrils are formed from monomeric peptides by primary nucleation. These fibrils are elongated by addition of monomeric peptides. Additionally, new aggregates are generated through secondary nucleation, where the formation of new nuclei is catalyzed by existing fibrils. This process has been shown to generate the majority of new nuclei for several Aβ species under a range of conditions, including in human cerebrospinal fluid (Cohen et al., 2013; Meisl et al., 2014, 2016a; Yang et al., 2018; Frankel et al., 2019). Secondary nucleation is observed for a wide range of self-assembling systems (Botsaris, 1976; Agrawal and Paterson, 2015; Anwar et al., 2015; Törnquist et al., 2018) and is composed of an arrival/binding step followed by a conversion/detachment step (Meisl et al., 2014). Depending on the relative rates of these processes, secondary nucleation can saturate above a certain monomer concentration, meaning that further increase in monomer concentration does not lead to a significant increase in the rate of secondary nucleation.

Here we use denaturants to alter the relative stabilities of monomeric and aggregated states to gain insights into the amyloid fibril formation mechanism and the determinants of the rates of the underlying steps. We examine how the different steps of the Aβ42 aggregation process are affected by denaturants through a combined experimental and theoretical approach. By utilising a natively unfolded peptide, we aim to advance the mechanistic understanding of the aggregation process without convolution with the unfolding step sometimes required for a natively folded protein. Moreover, in analogy with the larger stabilisation of unfolded relative to folded protein, which is coupled to the effect of denaturants on solubility, we may expect that denaturants stabilise various species along the aggregation pathway to a different extent thereby affecting the aggregation process in a non-trivial manner.

## Materials and methods

### Expression and purification of Aβ42

The amyloid β peptide M1-42 with sequence MDAEFRHDSGYEVHHQKLVFFAEDVGSNKGAIIGLMVGG VVIA (here referred to as Aβ42) was expressed in an *Escherichia coli* BL21 Gold (DE3) strain from a synthetic gene and purified using ion exchange and size exclusion as described elsewhere (Walsh et al., 2009).

### Preparation of Aβ42 samples for kinetic assays

Lyophilised Aβ42 peptide was dissolved in 1 mL 6 M GuHCl and monomeric peptide was isolated by size-exclusion chromatography in degassed buffer (20 mM sodium phosphate, 200 μM EDTA, 0.02% sodium azide, pH 8.0) on a Superdex 75 10/300 GL column (GE Healthcare). The center of the peak was collected, and the peptide concentration was determined by integrating the area under the absorbance curve using ε_280_ = 1,440 L mol^–1^ cm^–1^. The collected Aβ42 monomers were kept on ice and used the same day. Samples of 3 μM monomeric Aβ42 in presence of varying concentrations of urea, urea and NaCl and GuHCl were prepared. Additionally, dilution series of 1.6–5 μM Aβ42 in presence of 0.25 M, 1 M, 2 M, and 3 M GuHCl and 1.6–18 μM Aβ42 in the presence of 0.25 M, 1 M, 2 M, and 3 M urea were prepared. The wider range of peptide concentrations used in the presence of urea was motivated by the slower kinetics. All samples were prepared in low-binding tubes (Axygen) and supplemented with 6 μM thioflavin T (ThT, CalBiochem) from a concentrated stock that had been filtered through a 200 nm syringe filter.

### Kinetic assays

Eighty microliter of each sample was carefully pipetted into multiple wells of a 96-well half-area, low-binding, polyethylene glycol coated plate (Corning 3881), and the aggregation kinetics was studied by following the ThT fluorescence at 37°C under quiescent conditions in a plate reader (Fluostar Optima, Fluostar Omega, BMG Labtech). The ThT fluorescence was measured through the bottom of the plate using a 440 nm excitation filter and a 480 nm emission filter. Each experiment was repeated at least twice with 3–4 repeats of each sample.

### Kinetic assay with added seeds

Aβ42 seeds were prepared in buffer (20 mM sodium phosphate, 200 μM EDTA, 0.02% sodium azide, pH 8.0) without denaturant and with 0.25 M, 1 M, 2 M, or 3 M urea or 0.25 M, 1 M, or 2 M GuHCl at 37°C under quiescent conditions in a plate reader using ThT fluorescence as a validation of fibril formation. About 0.2–30% preformed seeds were added to 3 μM Aβ42, supplemented with 6 μM ThT, and the aggregation was followed as described above. The fraction of monomers in seeds at time zero is thus equal to (added amount of seeds)/(monomer + added amount of seeds). For technical reasons, investigations of the saturation of the elongation constant at high Aβ42 concentrations are difficult. Addition of seeds is necessary to investigate the effects on the elongation step alone, but at high Aβ42 concentrations addition of seeds results in very high aggregation rates, meaning the reaction is complete before sufficient data can be acquired.

### Theoretical analysis

The half time of aggregation (t_1/2_) is defined as the point where the ThT value is halfway between the initial baseline and the plateau value. The scaling exponent, γ, was obtained by plotting the t_1/2_ of each concentration against the initial monomer concentration (m_0_) on a double logarithmic plot and fitting a straight line to the data points:


(1) $$t_{1/2}\propto m_{0}^{\gamma }$$

The differential equations describing the time evolution of aggregate mass concentration, M(t), and aggregate number concentration, P(t) are


(2) $$\frac{d\,P}{d\,t}=k_{n}\,m\,{\left(t\right)}^{n_{c}}+k_{2}\,m\,{\left(t\right)}^{n_{2}}\,M\,\left(t\right)$$

for a single step nucleation process, or more generally


(3) $$\frac{d\,P}{d\,t}=k_{n}\,m\,{\left(t\right)}^{n_{c}}+k_{2}\,\frac{m\,{\left(t\right)}^{n_{2}}}{1+\frac{m\,{\left(t\right)}^{n_{2}}}{K_{M}}}\,M\,\left(t\right)$$

for a multi-step secondary nucleation process, along with


(4) $$\frac{d\,M}{d\,t}=2\,m\,\left(t\right)\,k_{+}\,P\,\left(t\right)$$

where k_*n*_, k_2_, k_+_ are the rate constants for primary nucleation, secondary nucleation and elongation, respectively. K_*M*_ is the saturation constant for secondary nucleation and n_2_ and n_*c*_ are the monomer scaling exponents (reaction orders) of primary and secondary nucleation, respectively. These models are used to fit the overall ThT fluorescence curves. Consequently, the rate constants are weighted averages over any conformations that are present in the fibrillar or monomeric state. An approximate solution to Equations 3, 4 is:


(5) $$\frac{\text{M}}{{\text{M}}_{\infty }}=1-\left(1-\frac{{\text{M}}_{0}}{{\text{M}}_{\infty }}\right)\,e^{-k_{\infty }\,t}*{\left(\frac{B_{-}+C_{+}\,e^{\kappa \,t}}{B_{+}+C_{+}\,e^{\kappa \,t}}*\frac{B_{+}+C_{+}}{B_{-}+C_{+}}\right)}^{\frac{k_{\infty }^{2}}{\kappa \,\alpha }}$$

where the parameters are defined by


(6) $$\kappa =\sqrt{2\,{\text{m}}_{0}\,k_{+}\,\frac{m_{0}^{n_{2}}\,k_{2}}{1+m_{0}^{n_{2}}/K_{M}}}$$

(7) $$\lambda =\sqrt{2\,k_{+}\,k_{n}\,m_{0}^{n_{c}}}$$

(8) $$C_{\pm }=\frac{k_{+}\,P_{0}}{\kappa }\pm \frac{k_{+}\,M_{0}}{2\,m_{0}\,k_{+}}\pm \frac{\lambda^{2}}{2\,\kappa^{2}}$$

(9) $$k=2\,k_{+}\,P_{\infty }$$

(10) $$\alpha =\sqrt{k_{\infty }^{2}-4\,C_{+}\,C_{-}\,\kappa^{2}}$$

(11) $$B_{\pm }=\frac{k_{\infty }\,\text{±}\,\alpha }{2\,\kappa }$$

where P_0_ is the aggregate number at the start of the reaction, P_∞_ is the aggregate number at equilibrium as described in Meisl et al. (2014), that is, after reaction completion, M_0_ is the mass concentration of fibrils at the start of the reaction and M_∞_ is the mass concentration of fibrils at equilibrium. The reaction order of secondary nucleation n_2_ = 2, as previously established, was consistent with all data. The reaction order for primary nucleation, n_*c*_ was fitted in the presence of denaturant and generally found to increase with denaturant concentration.

### Preparation of TEM samples

Monomeric Aβ42 peptide was isolated in 20 mM sodium phosphate buffer, 200 μM EDTA, 0.02% sodium azide, pH 8.0 by size-exclusion chromatography as described above. The sample was diluted with buffer and denaturant and ThT was added. The final concentrations were 1 M and 3 M of GuHCl or urea, or 1 M urea + 1 M NaCl, 9-10 μM Aβ42 and 1 μM ThT, pH 8.0. The aggregation kinetics were recorded using a Fluostar Optima plate reader (BMG Labtech) at 37°C under quiescent conditions. Samples for *cryo*-TEM were taken once the aggregation reaction had reached the fluorescence plateau. Five microliters were loaded on a lacey carbon filmed copped grid and flash frozen in liquid ethane at −180°C. The frozen samples were stored in liquid nitrogen and images were taken by a 120-kV electron microscope (Philips CM 120 BioTWIN Cryo). Alternatively, a Fischione Model 2550 cryo transfer tomography holder was used to transfer the specimen into the electron microscope (JEM 2200FS) equipped with an in-column energy filter (Omega filter), which allows zeroloss imaging. The acceleration voltage was 200 kV for the JEM2200FS electron microscope and zero-loss images were recorded digitally with a TVIPS F416 camera using SerialEM under low dose conditions with a 30 eV energy selecting slit in place.

### Surface plasmon resonance

The SPR experiments were performed using a Biacore3000 instrument (GE Healthcare), and CM3 sensorchips with carboxylic acid groups on short dextran chains. The four lanes of the sensorchip surface were activated with a mixture of 0.2 M 1-Ethyl-3-(3-dimethylaminopropyl)-carbodiimide and 0.05 M N-hydroxysuccinimide in water, followed by injection of short fibrils, ca 50 nm, produced by vigorous shaking of a fibrillar sample, at 0.5 μM in 10 mM NaAc, pH 4, to enable coupling of the fibrils via primary amines. The injection of fibrils led to an increase of ca. 3000 RU, after which the remaining functional groups on the sensorchip were reacted with 50 μl of 1 M ethanolamine, and the buffer flow was changed to 20 mM sodium phosphate, 200 μM EDTA, 0.005 % Tween20, pH 8.0. Monomers were added to grow the fibrils on the chip until a signal of ca. 20000 RU had been added. The sensorchip was then used to study monomer association and dissociation kinetics by injection of monomer at a series of concentrations, in flow buffer with 1, 2 or 3 M urea or 1 M GuHCl, after which the dissociation was followed during buffer flow with the same denaturant concentration as during the injection. For the data analysis, we solved the rate equations describing linear growth/dissociation of fibrils, with binding/dissociation of monomeric species to their surface. The relevant differential equations are given in (He et al., 1997), which are expanded to allow for two classes of binding sites at ends and sides of fibrils. The equations can be solved noting that during the dissociation phase m_0_ = 0 and during the association phase m_0_ = constant. In practice, because the amount of fibrils detected in the SPR measurement may vary between measurement cycles and between repeats, the concentration of bound monomer m_*b*_, is given by the differential equation


(12) $$m_{b}^{\prime }\,\left(t\right)=k_{a}\,m_{0}\,\left(\alpha \,\left(M_{0}+\left(k_{+}\,m_{0}-k_{o\,f\,f}\right)\,P_{0}\,t\right)-m_{b}\,\left(t\right)\right)-k_{d}\,m_{b}\,\left(t\right)$$

where α is the number of monomer binding sites per monomer in the fibril and k_*a*_ and k_*d*_ are the rate constants of monomer association and dissociation, respectively. M_0_, P_0_ and m_0_ denote the mass concentration of fibrils, the number concentration of fibrils and the monomer concentration. This equation takes into account the change in the length of the absorbed fibrils by addition (k_+_) or dissociation (k_*off*_) of monomer from the fibril ends. In the association part of the reaction this equation can be solved for m_*b*_(0) = 0, to give the equation that describes the baseline corrected SPR data as


(13) $$I=c_{1}\,\left(1-e\,x\,p\,\left(-\left(k_{d}+k_{a}\,m_{0}\right)\,t\right)\right)+c_{2}\,t$$

where c_1_ and c_2_ are constant dependent on the above constants and the conversion of bound concentrations to SPR signal. In the dissociation case we set m_0_ = 0 giving


(14) $$I=a_{1}\,\left(e\,x\,p\,\left(-k_{d}\,t\right)-1\right)-a_{2}\,t+a_{3}$$

where a_1_, a_2_ and a_3_ are constants and the conversion of bound concentrations to SPR signal. In both cases the parameters of interest are the association/dissociation rate constants in the exponential, k_*a*_ and k_*d*_.

### Monte Carlo simulations

In our computer model of secondary nucleation (Šarić et al., 2014), each protein particle is represented by a hard spherocylinder (see Figure 10A) and a protein can exist in three different conformations/states: (i) the unfolded state represents unfolded protein monomers that are able to bind to the fibril surface and aggregate into micellar-like oligomers by virtue of tip-to-tip non-specific interactions; (ii) the intermediate state which represents partially folded proteins that more readily assemble into oligomers but have a lower affinity for the fibril surface, and (iii) the β-sheet state that is able to assemble into compact elongated fibrillar structures due to strong directional interactions. The interconversion rates of these states are governed by their respective internal free energies. Nominally, we set the internal energies to *f*_*s*_ = 0 for the monomeric state, *f*_*i*_ = 1*kT* for the intermediate state, and *f*_β_ = *f*_*i*_ + 10*kT* for the β-sheet state. This introduces an energy penalty to forming a fibrillar aggregate from monomeric proteins, which can be overcome through a nucleation process. This nucleation process involves binding and oligomerisation of monomeric protein on the fibril surface, the conversion of surface-oligomers to more tightly bound intermediate oligomers, their detachment from the fibril surface, and finally a conversion of detached intermediate oligomers into compact, thermodynamically stable fibril nuclei.

We capture the influence of denaturant in our simulations by changing the internal energy of the monomeric state as *f*_*s*_→*f*_*s*_ + Δ*f*_*den*_, where Δ*f*_*den*_ < 0 represents the stabilising effect of the denaturant. More details can be found in our previous work (Šarić et al., 2016a).

We start our simulations with a preformed fibril at the middle of the simulation box and allow monomeric proteins to adsorb to the fibril until they reach a chemical equilibrium. After the equilibration phase we turn on the possibility of interconversions between different protein states. The rate of nucleation is recorded as the inverse of the first-passage time it takes for the fibril nucleus consisting of two mutually-interacting β-sheet proteins to be formed. Several simulations were run at different starting concentrations of the monomeric protein, allowing us to extract the reaction order and the average size of the critical nucleus at each value of Δ*f*_*den*_. For each set of simulation parameters, we ran 9 Monte-Carlo simulations at different random seed concentrations.

## Results

### Denaturant effect on fibril morphology

Before investigating the kinetics and mechanism of assembly, we determined the morphology of the formed fibrils and their ability to bind monomers on their surface, as a function of the denaturant concentration. To determine the fibril morphology, fibrils were formed from 9 to 10 μM Aβ42 monomers in 0.25, 1, 2, or 3 M urea or 0.25, 1, 2, or 3 M GuHCl and observed using *cryo*-TEM.

**FIGURE 1:**
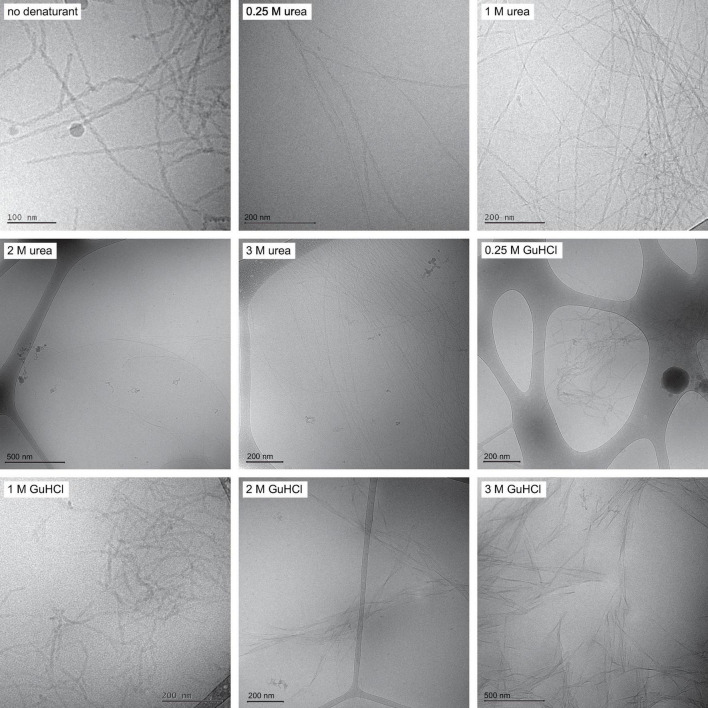
Fibril morphology *Cryo*-TEM images with fibrils formed in the absence of denaturant, in 0.25, 1, 2, and 3 M urea and in 0.25, 1, 2, and 3 M GuHCl. Fibrils formed in urea are on average longer than fibrils formed in buffer. Fibrils formed in 1 M GuHCl have similar morphology to fibrils formed in buffer but are on average shorter.

Fibrils formed in phosphate buffer with urea are found to be structurally similar to fibrils formed in phosphate buffer alone with filaments twisted around each other forming visible nodes in cryo-TEM images. Increasing urea concentration is observed to result in longer fibrils, suggesting a decrease of the nucleation rates relative to the growth rate (Figure 1 and Supplementary Figure 1). The fibrils formed in urea also appear more dispersed and evenly distributed over the grids than fibrils formed in phosphate buffer, which often occur as large dense aggregate clusters. Fibrils formed in phosphate buffer with GuHCl are on average shorter than fibrils formed in phosphate buffer (Figure 1). At 0.25 and 1 M GuHCl the overall appearance of the fibrils resembles the morphology of fibrils formed without denaturant present, displaying two filaments wound around each other and twisted to different degrees. The morphology of fibrils formed in 2 M GuHCl differs remarkably from fibrils formed in buffer, appearing more rigid, less twisted and sometimes containing more than two filaments. These images were used to estimate fibril length for analysis of the elongation rate constant k_+_ (Supplementary Figure 2). The appearance of fibrils formed in a solution containing both 1 M urea and 1 M sodium chloride resembles fibrils formed in 1 M GuHCl (Supplementary Figure 3).

### SPR analysis of surface adsorption in the presence of denaturant

**FIGURE 2:**
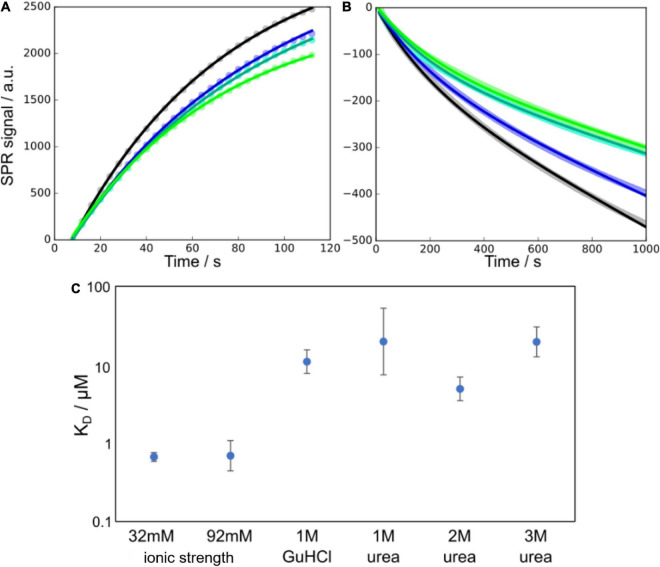
Monomer binding to fibrils as measured using SPR at different denaturant conditions. **(A,B)** Example SPR curves (points) and fits of Equations 13, 14 (solid lines), for the association **(A)** and dissociation **(B)** parts of the SPR curves at 1 M urea and 16 μM monomer concentration (four technical replicates). In panel **(B)** the data points are overlapping and appear as thick lines behind the fitted curves. **(C)** Affinities, in terms of the equilibrium dissociation constant K_*D*_, of monomers to the fibril surface, at a number of different denaturant concentrations. The data at 32 and 92 mM ionic strength are from Meisl et al. (2017a) and show the higher affinity in the absence of denaturant and the negligible effect that changes in ionic strength have in this range of salt concentrations.

The surface of Aβ42 fibrils plays an important role as a catalytic site for secondary nucleation. This process is believed to involve the adsorption of smaller Aβ42 species to the surface of fibrils, where nucleation takes place. In order to determine to what extent this adsorption is affected by the presence of denaturant, we performed surface plasmon resonance (SPR) experiments in the absence and presence of denaturant at several concentrations. Taking into account the growth and dissociation of fibrils, we were able to determine the rate constants for adsorption and dissociation of surface-bound species. The ratio of the dissociation and association rate constants was used to estimate the apparent equilibrium dissociation constant, K_*D*_. We find that K_*D*_ increases significantly in the presence of denaturant, thus reflecting a reduced affinity for the fibril surface, consistent with a stabilisation of the free over the bound state and decreased surface coverage, i.e., decreased number of surface-bound peptides per unit length of fibril (Figure 2). To also highlight the effect of the ionic strength of the solution on this affinity, which is likely to be important for low concentrations of GuHCl, we also show previously determined K_*D*_ values at high ionic strengths but in the absence of denaturant.

### Effects of urea and GuHCl on the Aβ42 aggregation rate

**FIGURE 3:**
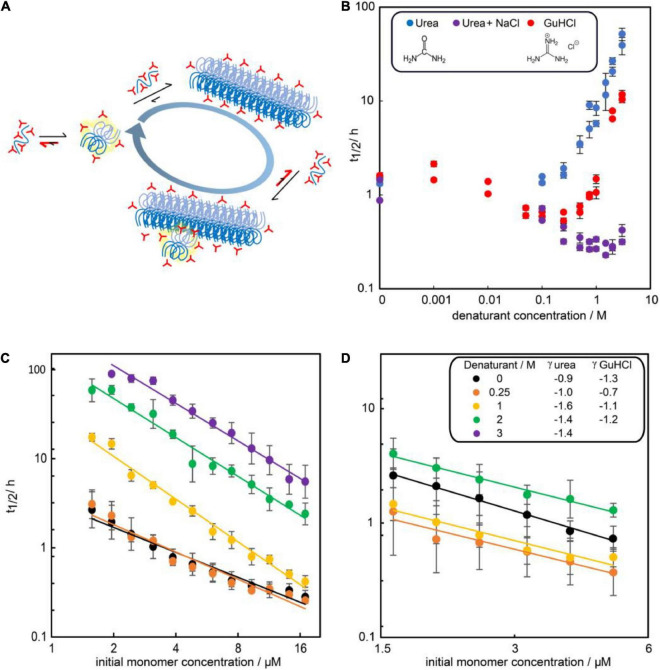
Influence of denaturants on the aggregation timescale of Aβ42. **(A)** Denaturants (red) favor smaller and less folded species in the reaction network more than large and folded species. **(B)** Half time of fibril formation versus denaturant concentration for solutions of 3 μM Aβ42 and urea (blue), GuHCl (red) or an equimolar mixture of urea and NaCl (purple) in 20 mM sodium phosphate, 0.2 mM EDTA, 0.02% sodium azide, pH 8.0. **(C)** Half time of fibril formation as a function of Aβ42 monomer concentration in the presence of no (black), 0.25 M urea (orange), 1 M (yellow), 2 M (green), or 3M (purple) urea. **(D)** Half time of fibril formation as a function of Aβ42 monomer concentration in the presence of no (black), 0.25 M (orange), 1 M (yellow), or 2 M (green) GuHCl. Each data point is the average of the log half time over at least two repeats of aggregation experiments with three replicates at each concentration and the error bars represents the standard deviations from all repeats. In panels **(C,D)**, the scaling exponents were estimated by fitting a power function to the data at each denaturant concentration (Equation 1).

To investigate the effect of the two denaturants on the kinetics, the aggregation of 3 μM Aβ42 was studied in 0–3 M urea or 0–3 M GuHCl (Figure 3). Aggregation in at least two independent experiments of triplicate repeats were monitored using ThT fluorescence. To quantify the aggregation propensity, the half time of aggregation is used, which is defined as the time by which half the final aggregate concentration has formed. We find that addition of urea monotonously decreases the aggregation rate of Aβ42 while addition of GuHCl increases the aggregation rate at low GuHCl concentrations and decreases the aggregation rate at GuHCl concentrations above 1 M. The maximum aggregation rate is reached at approximately 0.25 M GuHCl. This difference is likely to originate from the fact that GuHCl is charged, whereas urea is not. As we have shown previously (Meisl et al., 2017a), electrostatic shielding by charged molecules, in this case GuHCl, speeds up the rate of Aβ42 aggregation. Indeed, we find that aggregation in the presence of an equimolar mixture of sodium chloride and urea mimics the behaviour of GuHCl (Figure 3B).

### Effect of urea on the rate of Aβ42 aggregation and its monomer-concentration dependence

The aggregation of Aβ42 in presence of urea was followed by the ThT fluorescence intensity (Supplementary Figure 4), which was shown to be proportional to the total fibrillar mass (Supplementary Figure 5). The aggregation kinetics were studied at 0, 0.25, 1, 2, and 3 M urea over time at a range of protein concentrations (1.6–18 μM). All experiments started from supersaturated monomer solutions and were conducted at 37°C under quiescent conditions. These experiments showed decreasing ThT intensity with increasing urea concentrations, which was found not to be caused by any major increase in solubility, as confirmed by SDS-PAGE, but is instead believed to be an effect of altered ThT binding or fluorescence quantum yield (Supplementary Figure 6).

The dependence of the half time on the monomer concentration, described by the scaling exponent, provides insight into possible mechanisms of aggregation. The scaling exponent varies with urea concentrations (Figure 3C). Moreover, the scaling exponent shows some concentration dependence in the absence of denaturant, evident in the curvature of the double logarithmic plot of monomer concentration against half time. This curvature indicates that one or more of the microscopic steps involved in the aggregation process may become saturated at high concentrations of monomeric peptide (Meisl et al., 2017b). The reported scaling exponents are thus averages over the whole concentration range sampled. Upon addition of urea the scaling exponent decreases, i.e., becomes more negative with a higher absolute value. The decreased scaling exponent indicates that the monomer dependence of the Aβ42 aggregation process increases in the presence of urea.

**FIGURE 4:**
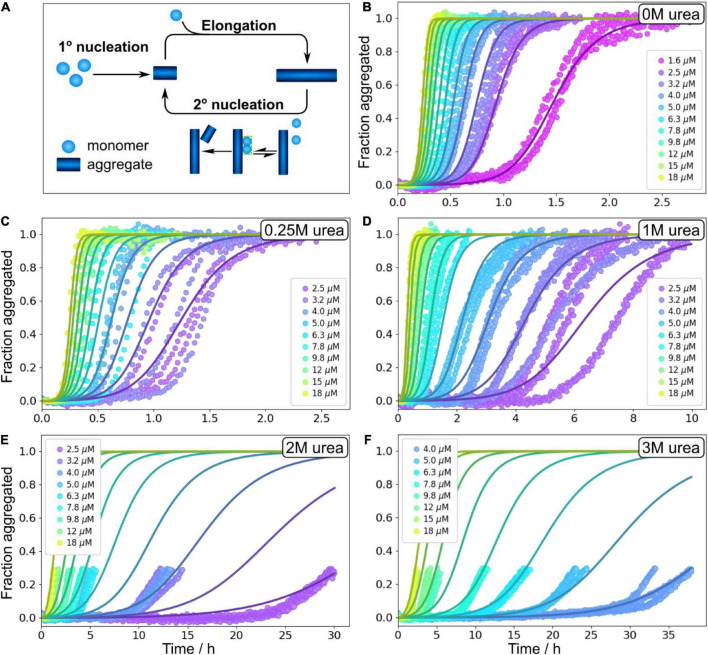
Global fitting of Aβ42 aggregation in urea. **(A)** Schematic aggregation reaction. Aβ42 aggregation is initiated by primary nucleation followed by elongation. New nuclei are also generated through secondary nucleation where an initial attachments step is followed by a conversion/detachment step. **(B–F)** Aβ42 aggregation data in 0 M urea, 0.25 M urea, 1 M urea, 2 M urea, and 3 M urea in 20 mM sodium phosphate, 0.2 mM EDTA, 0.02% sodium azide, pH 8.0 were fitted globally with a model that includes all the steps depicted in panel **(A)**, see Equation 5. At high urea concentration, we show here fitting to the onset of aggregation, and the full data sets are shown non-normalised in Supplementary Figure 4. Additional globally fitted data set is shown in Supplementary Figure 7.

In order to obtain quantitative mechanistic insights into the influence of urea on the Aβ42 aggregation process, we fitted the kinetic traces for Aβ42 in 0, 1, 2, and 3 M urea using the Amylofit platform (Meisl et al., 2016b). The kinetics are well fitted by the following model: Monomeric proteins nucleate in solution to form fibrils in the process of primary nucleation. These fibrils can then grow by the addition of monomeric proteins onto the end of existing fibrils in the process of elongation. Finally, existing fibrils can self-replicate by catalysing the formation of new nuclei on their surface via secondary nucleation. This secondary nucleation process consists of several steps in series with an initial attachment step to the fibril surface, followed by a rearrangement/detachment step (Figure 4A). Depending on the conditions, the relative balance between these steps can be shifted. When the catalytic fibril surface is fully covered, the overall behaviour is dominated by the rearrangement/detachment step, which we refer to as the saturated regime. When the surface is not fully covered, the overall rate also depends on the attachment step, which we refer to as the unsaturated regime. The aggregation of Aβ42 over a small range of monomer concentrations, in the absence of denaturant, can be explained by a model that operates exclusively in the unsaturated regime (Cohen et al., 2013). However, to explain the data obtained over a large range of Aβ42 concentrations and in the presence of denaturant the possibility of saturated secondary nucleation needs to be included. All kinetic curves at all peptide concentrations ranging from 1.6 or 2 μM to 18 μM (except for at 3 M urea where the fitted concentration range was 3.6–18 μM) were fitted by this model including primary nucleation, elongation and surface-catalyzed secondary nucleation (Figure 4 and Supplementary Figure 7). The parameters of the model are the rate constants of the three processes, elongation (k_+_), primary nucleation (k_*n*_) and secondary nucleation (k_2_), the concentration at which secondary nucleation is half saturated (K_*M*_^1/n2^), and the reaction orders of primary nucleation, n_*c*_, and secondary nucleation, n_2_. The data at all conditions is consistent with a multi-step secondary nucleation model with n_2_ = 2, in line with previous findings for Aβ42 aggregation in phosphate buffer (Cohen et al., 2013; Meisl et al., 2016a,2017a; Yang et al., 2018). The reaction order of primary nucleation has been previously determined to be n_*c*_ = 2 in the absence of denaturant and we here found it to increase above this value with increasing urea concentration.

We note that in aqueous solution, urea decomposes into ammonium and cyanate, which leads to carbamoylation of proteins by cyanate. The effect at early time points is likely to be minor, and we therefore focus our analysis on data up to maximum 38 h. At 2 and 3 M urea, the kinetic traces show a prolonged drift in the region where the ThT signal would normally plateau; therefore, normalisation and fitting of the data in the standard way was not possible. We instead focus on the initial part of these kinetic curves, before the half time is reached as data up to the half time generally give sufficient constraints to determine a mechanism and its rates, and this treatment avoids problems resulting from the drift behaviour. Because the possible molecular reasons for such a drift include sedimentation, clumping, gelation, etc. which are not part of our models, the traces past the initial sigmoidal part cannot be described by these models. The full data are shown in (Supplementary Figure 4).

### Seeded Aβ42 aggregation in the presence of urea

**FIGURE 5:**
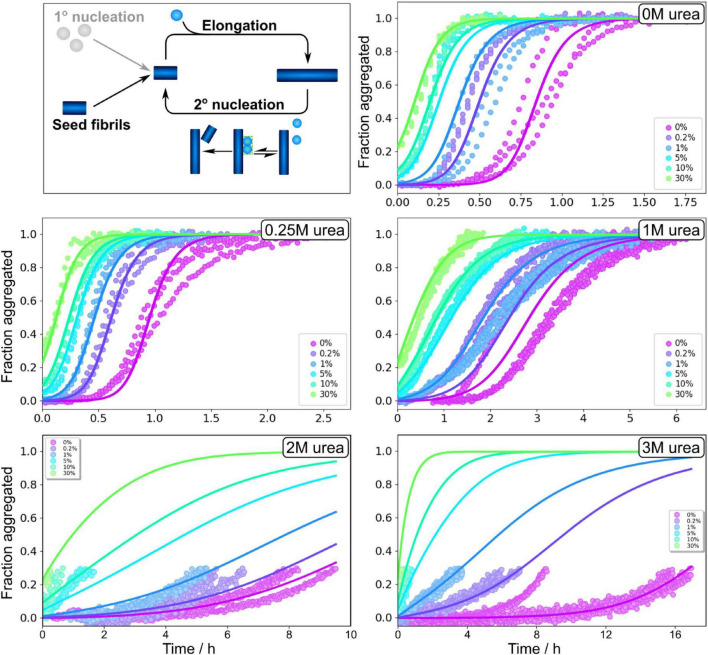
Seeding experiments in urea. Normalised ThT fluorescence as a function of time for 3 μM Aβ42 in buffer with no urea, 0.25 M urea, 1 M urea, 2 M urea, and 3 M urea in 20 mM sodium phosphate, 0.2 mM EDTA, 0.02% sodium azide, pH 8.0. Seeds, prepared at the corresponding urea concentration, were added at time zero at concentrations of 0.2, 1, 5, 10, and 30% in monomer equivalents. In the presence of a secondary nucleation step, small seed concentrations allow one to bypass the primary nucleation step. The solid lines show fits to the data using the same model as for the unseeded data with *n*_2_, *n*_*c*_ and *K*_*M*_, were fixed to the values determined in the unseeded conditions, whereas *k*_*n*_, *k*_+_ and *k*_2_ were globally fitted over the six seed concentrations.

Measurement of the total aggregate amount, as we obtain here from ThT measurements, only provides information on the combined rate constants of primary nucleation and elongation, k_+_k_*n*_, and of secondary nucleation and elongation, k_+_k_2_. The individual rate constants can be estimated using seeding experiments, although uncertainties on the values of these parameters are generally much larger than for the combined rate constants as the determination of the individual rate constants requires estimation of the average length of seed fibrils used, which is complicated by clumping of fibrils. Seeding experiments were performed for 3 μM Aβ42 monomers with preformed fibrils at 0, 0.2, 1, 5, and 30% in monomer equivalents (Figure 5 and Supplementary Figure 8). The seeds were prepared in solutions with the same concentration of urea as in the kinetic measurements that they were subsequently used in. We find that the half time is shortened by addition of preformed seeds at all urea concentrations studied. Even at low concentrations of preformed seeds, the seeding effect is strong, confirming that the dominant process generating new aggregates is indeed still secondary nucleation. The seeding propensity of a given amount of seeds increases with increasing urea concentrations, which is likely due to the lower abundance of densely packed fibril clusters within the seed aliquot, making more seed fibrils accessible for secondary nucleation the higher the denaturant concentration. The fits to the seeded data are generally of a similar quality to those of the unseeded data. As summarised in Supplementary information, the model used to fit all data is consistent both with the unseeded and seeded aggregation data and both types of data yield comparable rate constants.

**FIGURE 6:**
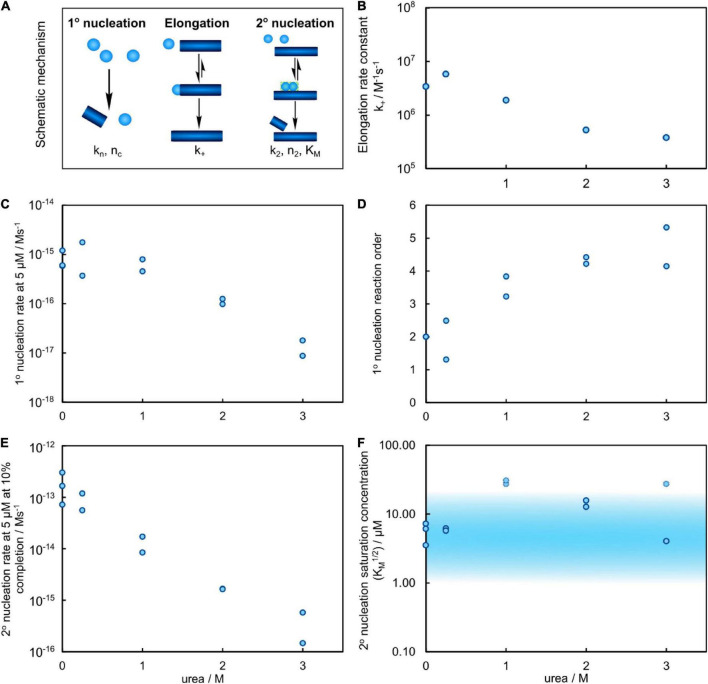
Results of the global fitting for the aggregation process of Aβ42 in urea. **(A)** Schematic of the individual processes of the aggregation reaction, with their associated parameters. **(B)** Estimation of the elongation rate constant from the cryo-TEM measurements and heavily seeded aggregation experiments versus urea concentration shows a decrease of the rate by approximately one order of magnitude at 3 M urea. **(C)** The primary nucleation rate versus urea concentration, evaluated for 5 μM Aβ42, decreases by approximately 2 orders of magnitude in 3 M urea. **(D)** The reaction order of primary nucleation, related to the associated nucleus size, shows a clear and significant increase with increasing urea concentration. **(E)** The secondary nucleation rate (for 5 μM Aβ42 at 10% completion), decreases by approximately 3 orders of magnitude. **(F)** The monomer concentration at which secondary nucleation becomes half saturated versus urea concentration. The shaded region marks values within the region of sampled monomer concentrations which are thus more well constrained. At 1 M urea the reaction is not significantly saturated, so only a lower bound on the saturation concentration can be obtained, as shown by the faint points.

The elongation rate constants were estimated from heavily seeded aggregation experiments and TEM measurements (Meisl et al., 2014) as outlined in (Supplementary Figures 1, 2). The elongation rate was found to vary by less than an order of magnitude for all urea concentrations investigated (Figure 6). Thus, the reduced aggregation rate caused by addition of urea originates primarily from decreased rates of primary and secondary nucleation.

As the secondary nucleation rate saturates at different concentrations depending on the urea concentration, the actual rate of formation of secondary nuclei, rather than the rate constant, is the most meaningful quantity to compare. Thus, the predicted rates of secondary nucleus formation at 10% conversion of an initially monomeric sample, at a total monomer concentration of 5 μM, are computed from the fitted rate constants and shown in Figure 6E. Increasing urea concentration results in decreased rate of secondary nuclei generation. Because the reaction orders change with urea concentration, we also show the rate, rather than rate constant, of primary nucleus formation at 5 μM monomer concentration in Figure 6C. Also in this case, the rate decreases with increasing urea concentration, by approximately two orders of magnitude between 0 and 3 M urea.

### Effect of GuHCl on the aggregation of Aβ42

Aβ42 aggregation kinetics of 1.6–5 μM Aβ42 in the presence of 0, 0.25, 1, 2, and 3 M GuHCl was studied by monitoring the ThT fluorescence intensity (Supplementary Figure 9), which was established to be proportional to the fibrillar mass at GuHCl concentrations <3 M (Supplementary Figure 5). At low GuHCl concentrations (0.25 and 1 M) the overall aggregation rate of Aβ42 is accelerated whereas at high GuHCl concentrations (2 and 3 M) the overall aggregation rate is decreased. 3 M GuHCl is experimentally difficult to work with, markedly reduces the ThT signal intensity, and the altered morphology of the fibrils results in difficulties in measuring the fibril length which is needed for any additional analysis. Thus, the aggregation experiments in 3 M GuHCl were not used in the following more quantitative analysis.

The non-monotonic aggregation behaviour in GuHCl contrasts with the effect of urea, and, as discussed later, is likely due to the charged nature of GuHCl. While at high GuHCl concentrations the scaling exponent is the same as in the absence of GuHCl, at intermediate GuHCl concentrations, the scaling exponent increases, i.e., it becomes less negative (Figure 3D). This indicates that the aggregation rate is less monomer dependent.

**FIGURE 7:**
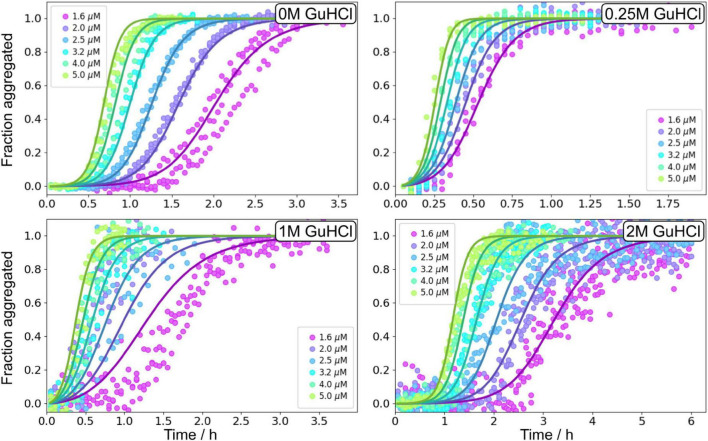
Global fitting of Aβ42 aggregation in GuHCl. Aβ42 aggregation in the absence of GuHCl, at 0.25, 1, and 2 M GuHCl in 20 mM sodium phosphate, 0.2 mM EDTA, 0.02% sodium azide, pH 8.0 were fitted with a model describing secondary nucleation as a multistep event, see Equation 5. Additional globally fitted data set is shown in Supplementary Figure 10.

**FIGURE 8:**
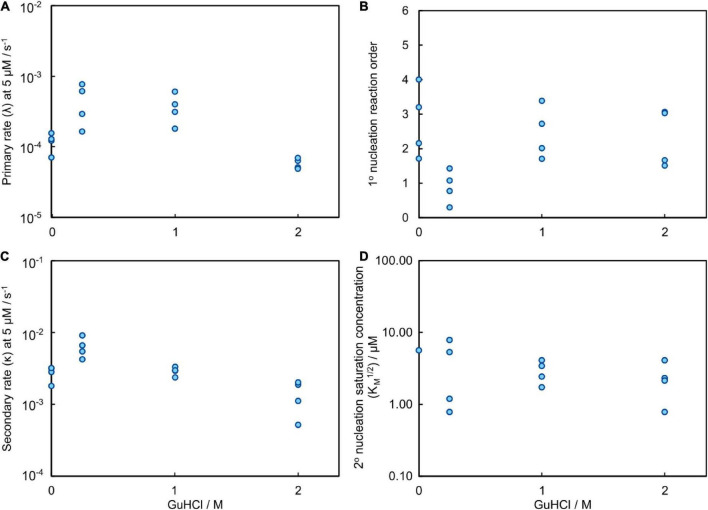
Results of the global fitting for the aggregation process of Aβ42 in GuHCl. **(A)** The rate at which fibrils are formed via the primary nucleation pathway, λ, evaluated at 5 μM Aβ42; it contains contributions from both elongation and primary nucleation (detailed definition see methods Equation 7). **(B)** The reaction order of primary nucleation shows a marked dip at 0.25 M GuHCl. The reaction orders are less well constrained than for the data obtained with urea because a smaller range of Aβ42 concentrations was sampled in the presence of GuHCl. **(C)** The rate at which fibrils are formed via the secondary nucleation pathway, κ, evaluated at 5 μM Aβ42; it contains contributions from both elongation and secondary nucleation (detailed definition see methods Equation 6). **(D)** The monomer concentration at which secondary nucleation becomes half saturated. The saturation effect on the aggregation process decreases slightly in the presence of denaturant, however, the exact values are less well constrained than for the data obtained with urea because a smaller range of Aβ42 concentrations was sampled in the presence of GuHCl.

Global analysis using Amylofit (Meisl et al., 2016b) allows us to describe all kinetic data at all concentrations for Aβ42 aggregation in GuHCl using a multistep secondary nucleation model with n_2_ = 2 (Figure 7 and Supplementary Figure 10). Although, as previously demonstrated, Aβ42 aggregation in the absence of denaturant is well fitted by a model describing secondary nucleation as a one-step event for Aβ42 concentrations between 1 and 5 μM, for consistency we use here the same, more general model as for urea. This model is able to describe the data at all denaturant concentrations. The different morphology of fibrils prepared in GuHCl makes it difficult to measure the length of individual fibrils and consequently, the elongation rate constant and thus also the other individual rate constants could not be determined accurately. Still, the combined rate constants; k_+_k_*n*_ and k_+_k_*n*_, can be determined and show increased rates at low concentrations and decreased rates at 2 M GuHCl (Figure 8).

The parameter that governs when the initial step in secondary nucleation, i.e., where monomers or preformed oligomers attach to the fibril, becomes half saturated, K_*M*_^1/n2^, can only be determined accurately when it lies inside the studied concentration range. Otherwise only an upper or lower bound on its value can be given. We observe no or little signs of saturation of the secondary nucleation for Aβ42 aggregation in buffer without GuHCl in the investigated concentration range (1.6–5 μM). Thus, we can only obtain a lower bound and conclude that K_*M*_^1/n2^ ≥ 5 μM, which correlates well with the K_*M*_ obtained from the wider concentration range investigated in the urea datasets above. The secondary nucleation of Aβ42 aggregation in 0.25 M GuHCl is fully saturated in the investigated concentration range. Thus, we can conclude that K_*M*_ lies below the investigated concentration range, i.e., K_*M*_^1/n2^ < 1.6 μM. The obtained value from the fit was K_*M*_ ≈ 0.45 μM^2^ (√K_*M*_ = 0.67 μM) and was used to calculate the secondary nucleation rate. The secondary nucleation of Aβ42 aggregation in 1 and 2 M GuHCl saturates within the studied peptide concentration range, thus K_*M*_ can be determined accurately. (Figure 8D).

### Seeded Aβ42 aggregation in the presence of GuHCl

**FIGURE 9:**
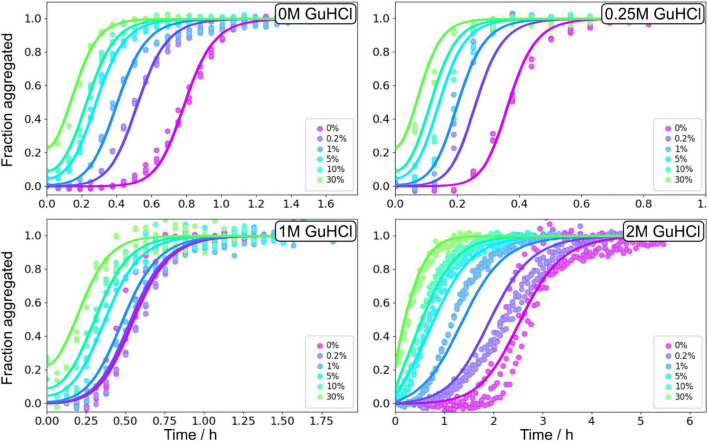
Seeding experiments in GuHCl. Normalised ThT fluorescence as a function of time for 3 μM Aβ42 in buffer, 0.25 M GuHCl, 1 M GuHCl, and 2 M GuHCl in 20 mM sodium phosphate, 0.2 mM EDTA, 0.02% sodium azide, pH 8.0. The samples were supplemented at time zero with seeds, prepared at the corresponding GuHCl concentration, at 0.2, 1, 5, 10, and 30% in monomer concentration units. The solid lines show fits to the data using the same model as for the unseeded data with *n*_2_, *n*_*c*_, and *K*_*M*_, were fixed to the values determined in the unseeded conditions, whereas *k*_*n*_, *k*_+_, and *k*_2_ were globally fitted over the six seed concentrations.

Seeded aggregation experiments were performed at 3 μM Aβ42 monomers with addition of preformed fibrils at 0, 0.2, 1, 5, and 30% in monomer equivalents (Figure 9). The seeds were prepared under the same conditions, i.e., the same GuHCl concentration, that they were subsequently used in. Seeding experiments confirmed that the main source of new nuclei is again secondary nucleation since the addition of small amounts of seeds shortened the lag phase at all denaturant concentrations.

**FIGURE 10:**
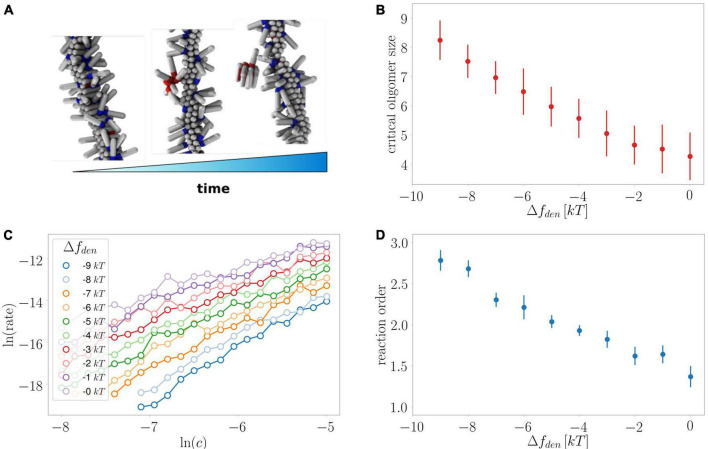
Monte Carlo simulations of secondary nucleation. **(A)** Depiction of the formation and detachment of a secondary nucleus of critical size as simulated in the current study. **(B)** Average critical nucleus size versus the effect of denaturant on the free energy of the protein in the unfolded monomeric state. **(C)** The rate of secondary nucleation versus monomer concentration for a number of effects on the free energy of the monomeric protein. **(D)** The reaction order determined from the data in panel **(C)** versus the effect of denaturant on the free energy of the protein in the unfolded monomeric state. Oligomer size as well as reaction order are larger when the stabilising effect of denaturant on the unfolded state is largest.

### Monte Carlo simulations

We previously investigated the processes of primary and secondary nucleation using Monte Carlo simulations of a minimal computer model system of amyloid formation (Šarić et al., 2014, 2016a). In line with what we observe here for the effects of denaturants on primary nucleation, and consistent with classical nucleation theory (CNT), we previously found using simulations that a relative decrease in the free energy gain upon formation of the condensed phase resulted in increased nucleus sizes (Šarić et al., 2016b). For secondary nucleation, dissection of the effects that lead to an increased monomer dependence at higher denaturant concentrations is less straightforward as the degree of saturation of the fibril surface, in addition to the size of the converting/detaching species, affects the monomer dependence of the reaction. Here, our global fitting procedure was able to describe all experimental data assuming the reaction order remains unchanged and only the coverage varies (Figures 4–8), which was motivated by the finding by SPR of reduced affinity of monomers for the fibril surface in the presence of denaturant. However, it is still possible that there is a contribution from an increase in reaction order in the presence of denaturant.

We therefore utilised our minimal computer model system to investigate the effect of a stabilisation of the monomeric state at constant coverage for a range of monomer concentrations (Figure 10). Indeed, we find an increase in the reaction order the larger the energy difference between the monomeric and the aggregated state due to the presence of denaturant. Moreover, these simulations allow us to directly extract the nucleus size, which is found to increase along with the reaction order, confirming that both a decreased coverage and increased nucleus size are expected effects of the presence of denaturant.

## Discussion

Both intrinsically disordered polypeptides and globular folded polypeptides may form amyloids associated with disease. The current results can be understood by considering the different chemical nature of the two denaturants used and their differential effects on unfolded versus folded polypeptide and or aggregated species of various sizes and structures (Figure 3A). Denaturants promote unfolding of a monomeric protein since they favour the unfolded state more than the folded state; indeed, the unfolded state of a protein is expected to be favoured over the oligomeric state, which in turn is expected be favoured over the fibrillar state. Preferential stabilisation of the monomeric state will result in an increase in peptide solubility. Depending on whether the monomeric protein or peptide is folded or unfolded, the denaturants may also affect the overall aggregation kinetics differently. Aggregation kinetics of folded proteins can be accelerated by the presence of denaturants if fibrils form from the partially or fully unfolded state. The aggregation-prone regions are most often buried within the folded protein core and fibril assembly is slow either because the concentration of aggregation-prone unfolded monomer is so low that a nucleation event is very unlikely to take place or because the unfolding step itself is rate-limiting. The fibril formation rate may therefore increase at conditions that promote a significant population of unfolded/partially unfolded proteins, although such conditions do also increase the solubility of the protein, an equilibrium property. By contrast, for natively unfolded proteins, addition of denaturant does not increase the concentration of aggregation-prone monomer, but simply stabilises the unfolded monomeric form relative to the folded fibrillar form. While this leads to increased solubility, it also commonly leads to decreased rates of aggregation. This is because the transition states of the rate-determining steps in the assembly process are likely assembled, rather than monomeric structures. Therefore, the presence of denaturant increases the stability of the monomeric state relative to the transition states, decreasing the aggregation rate. This reasoning parallels that of a phi value analysis of protein folding (Dagget and Fersht, 2000).

The system studied here belongs to the latter category in that monomers of the small peptide Aβ42 are unfolded in solution. Indeed, our data show that the addition of urea retards the aggregation process of Aβ42. The detailed kinetic experiments and analyses show that the reduced aggregation rate is primarily an effect of reduced nucleation rates.

### Urea affects the saturation of the secondary nucleation

Apart from variations in fibril length and clumping, the fibrils formed in urea appear to have the same morphology as those formed in buffer. Based on the kinetic data we may infer that the addition of urea causes a change in the underlying mechanism by shifting the relative importance of the microscopic steps involved in the aggregation process. This is reflected by the scaling exponent that decreases (i.e., becomes more negative) in the presence of urea, indicating that the monomer dependence of the nucleation processes increases. The scaling exponent of secondary nucleation is approximately given by γ = −(1+n_2_)/2 when the process is unsaturated and approaches γ = -1/2 as it saturates. Thus, the observed decrease of the scaling exponent upon addition of a denaturant could reflect either a change in the degree of saturation or a increase in the reaction order n_2_, where the latter may be the result of a change in nucleus size. Indeed, both scenarios are consistent with the denaturant lowering the free energy of the monomeric state, more than other states; however, the orthogonal measurements of affinity for the fibril surface obtained by SPR show that the affinity of monomers for the fibrils surface becomes lower (i.e., K_*D*_ becomes higher) in the presence of denaturant implying that a decrease in surface coverage at all monomer concentrations below saturation is a significant contribution to the change in monomer dependence of the aggregation reaction. In line with this finding, the kinetic data at all denaturant concentrations are well described by varying degree of saturation of secondary nucleation and a reaction order n_2_ = 2 that is independent of the urea concentration. While a variation of fibril coverage, as confirmed by SPR, is sufficient to explain all data, we cannot exclude that a small increase in reaction order also occurs in the presence of urea. Therefore, we confirmed that a decrease in the free energy of the monomeric state at constant coverage indeed increases the nucleus size by minimal computer simulations of secondary nucleation, as discussed below.

### Reduced nucleation rates in presence of urea

The microscopic steps expected to be most affected by the addition of urea are those for which the reactants and transition state/products are most differentially affected by the denaturant. In the case of Aβ42, these are likely processes involving a transition from a denaturant accessible, disordered state, to a more compact one, in particular processes in which several species undergo such a transition. Indeed, both the primary nucleation rate and the secondary nucleation rate decrease by more than two orders of magnitude over the investigated urea concentration range. Those nucleation steps both involve more than one peptide changing from disordered to more compact, whereas the effect on the elongation rate, which is believed to only involve a single monomeric species interacting with an already ordered fibril end, is minor. Since the saturation of secondary nucleation varies with increasing urea concentrations, we compare the rate of formation (i.e. the sum of conversion and detachment) of a new secondary nucleus at a specific stage of the nucleation process, i.e., at 10% conversion to fibrils for reactions starting from 5 μM Aβ42 monomer (as described in the result section). In this calculation we assume that 90% of the monomers, i.e., 4.5 μM, are free and available to form new nuclei. The rate of this process is found to decrease by about 3 orders of magnitude between 0 and 3 M urea.

### Competing effects of GuHCl

Guanidinium hydrochloride is a salt as well as a denaturant, which results in two opposing effects on the aggregation rate, with an acceleration at low GuHCl concentrations and a deceleration at high GuHCl concentrations. On the one hand, addition of GuHCl increases the ionic strength which results in reduced electrostatic repulsion between charged molecules, on the other hand we expect similar denaturing effects as for urea. As monomeric Aβ42 has a net charge between -3 and -4 at pH 8.0, addition of ionic molecules, such as GuHCl, to the solution causes increased electrostatic screening between the monomers and between the monomers and fibrils. We have previously investigated the effects of modulation of the electrostatic interactions in detail, revealing an overall increase in the aggregation rate with increasing ionic strength and that the secondary nucleation rate is fully saturated (making the monomer-independent conversion/detachment step the rate determining step) at ionic strengths above 0.2 M (Meisl et al., 2017a). Indeed, in the current data, the rates of both secondary and primary nucleation increase in the presence of 0.25 M GuHCl. However, at GuHCl concentrations above 0.25 M GuHCl, when electrostatic interactions are fully screened, the rates again decrease, in a similar manner to that observed for urea. At 2 M GuHCl the fibril morphology appears to be altered, meaning that detailed mechanistic conclusions may not be drawn from the aggregation data at the highest concentrations of GuHCl. Still, a strong retarding effect of 2M GuHCl can be deduced from the reduced values of the combined rate constants; k_+_k_*n*_ and k_+_k_*n*_ (Figure 8).

The competing effects of the ionic denaturant GuHCl, resulting in acceleration at low concentrations followed by deceleration of the aggregation rate as the GuHCl concentration increases, can be mimicked by an equimolar mixture of sodium chloride and urea to the solution, see Figure 3. Urea is a weaker denaturant than GuHCl, which is reflected in the higher maximum aggregation in the presence of urea/NaCl, but the qualitative behaviour caused by GuHCl is recovered. The fact that the effects of guanidine hydrochloride are not fully recapitulated by a mixture of urea and NaCl might be due to guanidine hydrochloride being a stronger denaturant than urea, or the Hofmeister effect of ions on peptide hydration.

### Increased reaction order of primary nucleation

Secondary nucleation dominates the production of new nuclei for the majority of the reaction time-course and therefore the parameters associated with this process can be determined more accurately than those associated with primary nucleation. Nonetheless, we find that a significant increase in the reaction order of primary nucleation is required to fit the experimental data well. The reaction order in the absence of denaturant is approximately n_*c*_ = 2 and at 3 M urea this has increased to n_*c*_ = 5. While the reaction order of this coarse-grained nucleation step is usually not directly equivalent to a nucleus size, its increase is likely to reflect an increase in the nucleus size. Indeed, simple classical nucleation theory (CNT) predicts an increase in the nucleus size as the nucleation barrier between the monomeric and aggregated states of the protein increases due to a stabilisation of the monomeric state by denaturant. The simulations using a minimal computer model (Figure 10) also recover such changes in nucleus size.

## Conclusion

Denaturants have a stronger effect on the unfolded relative to folded states of a protein, thereby shifting the equilibria towards the unfolded state and likely increasing the energy difference between the unfolded monomeric state and the more compact transition states of aggregation. For an intrinsically disordered protein, such as Aβ42, the addition of denaturant thus impedes the transition from unfolded monomers to the more compact oligomers and ordered fibrils through stabilisation of the monomeric over the aggregated states. The presence of denaturant differentially affects the rates of the individual steps in the aggregation process and slows the aggregation of Aβ42 mainly by decreasing the rates of the nucleation processes, with only a minor effect on the elongation of fibrils. The observed effect of urea on secondary nucleation of Aβ42 may be explained by a decrease in both the rate constant of nucleus formation and in the degree of surface saturation, both of which are due to stabilisation of the free over the bound monomeric state by denaturant. The combined effect is a decrease of the secondary nucleation rate by over two orders of magnitude. The rate of primary nucleation decreases by a comparable amount and its reaction order was found to increase upon addition of urea, again consistent with a stabilisation of the solution state relative to the nucleated state. While the increase in monomer dependence of the aggregation reaction, which we observe in the kinetic experiments in the presence of urea, can be accounted for fully by a decreased saturation of the fibril surface during secondary nucleation, simulations suggest that an increase in the secondary nucleus size may also contribute.

The ionic denaturant GuHCl combines two opposing effects on the Aβ42 aggregation process. Electrostatic screening of the repulsion between monomers and between monomers and fibrils leads to acceleration of aggregation, which dominates at low GuHCl concentrations. Equivalently to urea, the denaturing effect results in reduced aggregation rates and dominates at high GuHCl concentrations. Indeed, this behaviour can be mimicked using a mixture of urea and NaCl.

Our findings show that the stabilisation of the unfolded monomeric state by addition of denaturant has wide reaching effects on the diverse steps of the protein aggregation network: not only is the equilibrium solubility of the protein increased, but most reaction rates are also affected, likely because the transition states of the rate-determining steps are more compact and folded than the aggregation-prone monomer. Generally, the processes that involve the interaction of several monomers, primary and secondary nucleation are most affected. These effects take the form both of increases in nucleus sizes and decreases in the coverage of surfaces that catalyse nucleation. These results highlight the richness of behaviour that can result from a simple change in free energy of the monomeric state in protein aggregation networks.

## Data availability statement

The raw data supporting the conclusions of this article will be made available by the authors, without undue reservation.

## Author contributions

All authors listed have made a substantial, direct, and intellectual contribution to the work, and approved it for publication.
