# Supplementary material for: Influence of denaturants on amyloid β42 aggregation kinetics
Source: Front Neurosci. 2022 Sep 20;16:943355. doi: 10.3389/fnins.2022.943355 (PMC9531139; doi:10.3389/fnins.2022.943355)
Supplement: Supplementary file 1 [file Data_Sheet_1.pdf]

Supplementary information for

Influence of denaturants on amyloid β42 aggregation kinetics

SI Methods

Estimation of the elongation rate constants

Elongation rate constants were estimated from strongly seeded aggregation experiments, with Aβ42 monomer concentrations ranging from approximately 2 to 5 μM for measurements performed in urea. Seed concentrations were 3 μM for experiments performed in 0, 1 and 2 M urea and 5 μM for experiments performed in 3 M urea. All Aβ42 seeds were prepared at the same concentration of denaturant as they were subsequently used in. The initial gradient, $dP/dt|_{t=0} = 2k_+P_0m(t)$, at different monomer concentrations was used to extract $2k_+P_0$. The initial fibril number concentration, P_0, has to be assessed to obtain an estimation of the elongation rate constant, k_+. This was achieved by estimating the average number of monomers per seed fibril, L_0 (Fig. S1), which relates P_0 to initial fibril mass concentration, M_0, by $P_0 = M_0/L_0$. M_0 is known and L_0 was estimated from fibril dimensions measured in cryo-TEM images. The number of monomers per seed fibril was calculated based on the measured fibril dimensions, a molecular weight of 4.65 kDa per Aβ42 and assuming a density of peptides of 1.3 g/mL (Fig. S1). The fibril dimensions were assessed by measuring the end-to-end distance and width of individual fibrils using ImageJ. Due to the difficulties in obtaining exact fibril lengths, with larger fibrils being harder to measure, the average fibril dimensions are likely to be very approximate. Together with the number of monomers per fibril the elongation rate constants for Aβ42 aggregation in urea were estimated (Fig. S1C) from the initial slopes of the heavily seeded aggregation experiments (Fig. S2). Ionic GuHCl screens the electrostatic repulsion between fibrils. Thus, fibrils formed in GuHCl tend to cluster together which makes it difficult to measure the end-to-end distance of individual fibrils and consequently can the elongation constant, k_+, not be estimated. A very rough estimate of the fibril length suggests that fibrils formed in 1 M GuHCl are on average shorter and thicker than fibrils formed in buffer (300 x 70 nm for fibrils formed in 1 M GuHCl compared to 760 x 54 nm for fibrils formed in buffer).

Heavily seeded aggregation experiments also serve to evaluate possible saturation of the elongation rate. Saturation of the elongation rate results in non-linear increase of the initial gradient with monomer concentration and reaches a plateau at high concentrations. Aggregation experiments performed in urea showed linear increase of the initial gradient at all concentrations measured (2-5 μM Aβ42, 0-3 M urea), thus showed no indication of saturation of the elongation rate constant within this concentration range (Fig. S2A). Heavily seeded aggregation experiments performed in GuHCl showed linear increase of the initial gradient for GuHCl concentrations 0.25 – 2 M (Fig. S2B). The seed concentrations were 2 μM for aggregation experiments performed in 0.25 M GuHCl, 3 μM for experiments performed in 1 M GuHCl and 4 μM for experiments performed in 2 M GuHCl. It was technically very hard to obtain data with sufficient quality to study the initial aggregation slope of Aβ42 aggregation in 3 M GuHCl, thus this concentration was excluded from this experiment.

SI results – the analysis of data from seeded experiments

In order to determine whether the seeded data are consistent with the same model as the unseeded data, we fitted the seeded data with the same model as the unseeded data and 3 free fitting parameters (k_n, k_+ and k_2). As the seeded data were only recorded at a single monomer concentration, the parameters that can only be accurately determined from multiple monomer concentrations, n_c and K_M, were fixed to the values determined in the unseeded conditions. The rates of λ and κ, as given in equations 6 and 7, are used to compare the rate constants obtained in the seeded and unseeded experiments. For κ, a measure of secondary nucleation, the rates are within a factor of 3 for the systems for all denaturant concentrations. For λ, a measure of primary nucleation, the rates are within a factor of 5 of each other. The fits to the seeded data are generally of a similar quality to those of the unseeded data. In conclusion, the model is consistent both with the unseeded and seeded aggregation data and both types of data yield comparable rate constants.

Table S1. Ratios (unseeded over seeded) of λ and κ, evaluated at 3 μM, using the values of the rate constants obtained in the fits of the unseeded data and seeded data in Fig. 6.

**Urea**	**κ ratio**	**λ ratio**
0 M	0.55	2.01
0.25 M	0.61	2.03
1 M	0.57	0.45
2 M	0.60	0.21
3 M	0.32	0.35

Table S2. Ratios (unseeded over seeded) of λ and κ, evaluated at 3 μM, using the values of the rate constants obtained in the fits of the unseeded data and seeded data in Fig. 9.

**GuHCl**	**κ ratio**	**λ ratio**
0 M	0.43	2.23
0.25 M	0.52	4.61
1 M	0.70	0.73
2 M	1.27	0.82

A

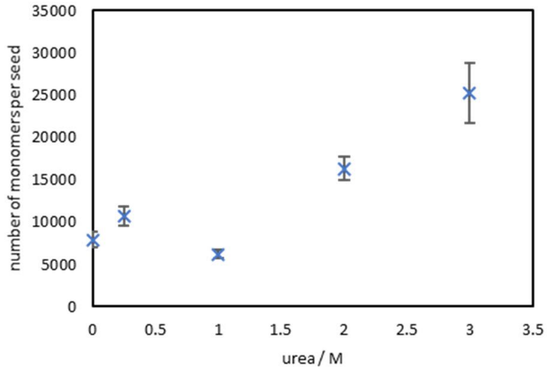

B

Denaturant / M	length / nm	cross area / nm^2	monomers/seed
0	760	54	7900
0.25	1000	58	10800
1	900	40	6200
2	1600	57	16000
3	3000	39	25000

C

Denaturant / M	k_+ in urea $M^{-1}s^{-1}$
0	$3.4 \cdot 10^6$
0.25	$5.8 \cdot 10^6$
1	$1.9 \cdot 10^6$
2	$5.3 \cdot 10^5$
3	$3.8 \cdot 10^5$

Figure S1. Fibril dimensions determined from *cryo*-TEM images. (A) The fibril dimensions vary with increasing denaturant concentrations. The error bars shown are the standard errors of the mean. (B) Addition of urea to Aβ42 peptides results in longer fibrils with similar cross area. (C) Estimations of the elongation rate constants at varying denaturant concentrations.

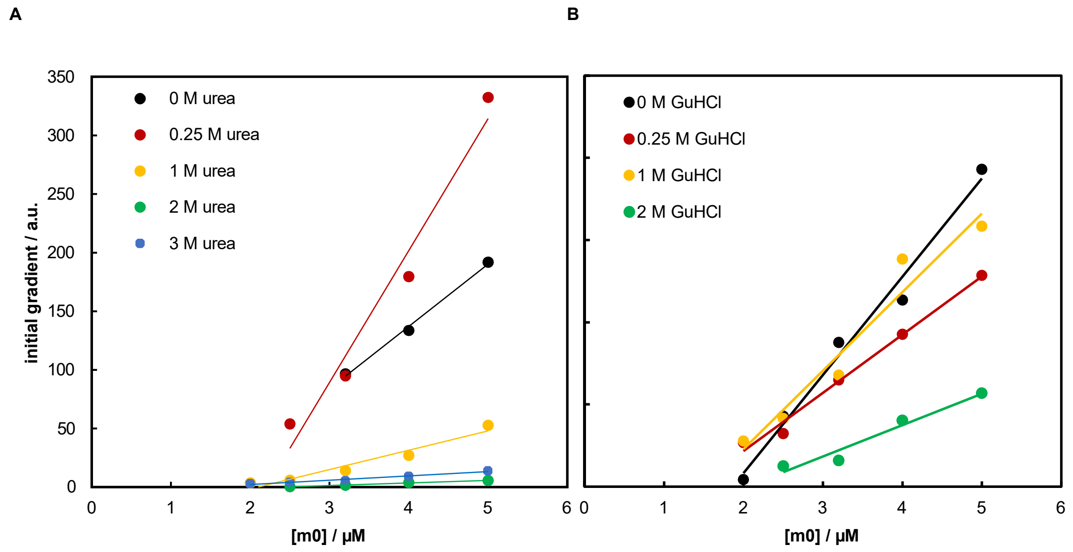

Figure S2. Initial gradients from strongly seeded Aβ42 aggregation experiments in denaturants. (A) The initial gradients for Aβ42 aggregating in presence of urea show a linear dependence on the monomer concentration implying that there is no saturation of the elongation rate at Aβ42 concentrations below 5 µM. (B) A typical curve for the initial gradients for Aβ42 aggregating in presence of 0.25 – 2 M GuHCl displaying a linear dependence on the monomer concentration indicating that there is no saturation of the elongation rate at Aβ42 concentrations below 5 µM.

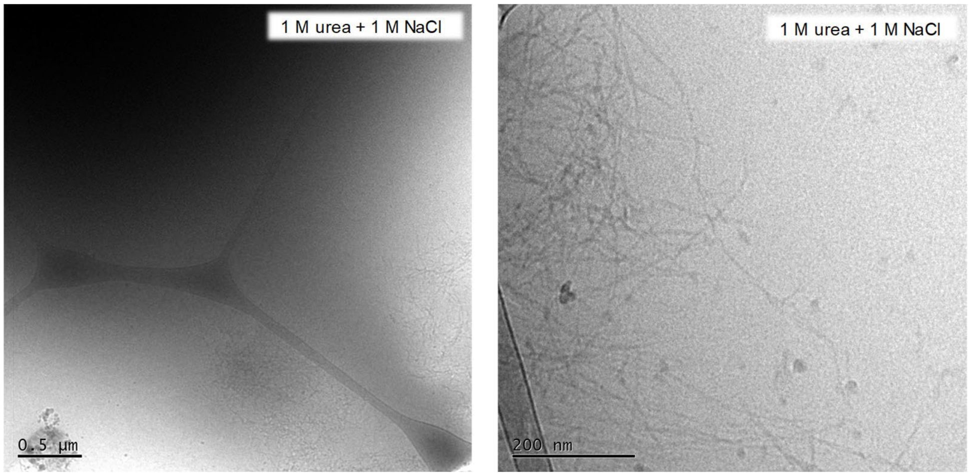

Figure S3. Aβ42 fibrils formed in 1 M urea and 1 M NaCl images by cryo-TEM.

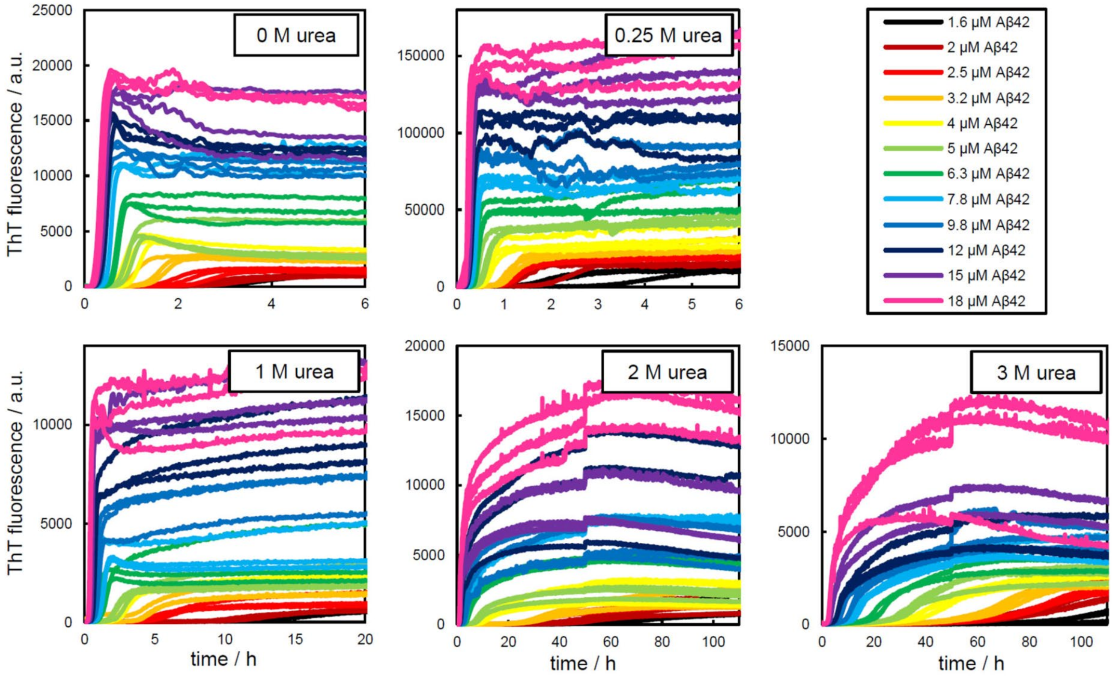

Figure S4. Concentration dependent aggregation of Aβ42 in urea. The aggregation process of Aβ42 was monitored by ThT fluorescence intensity as a function of time at varying urea concentrations. Different gains were set on the plate reader in presence and absence of urea since the signal was quenched in urea.

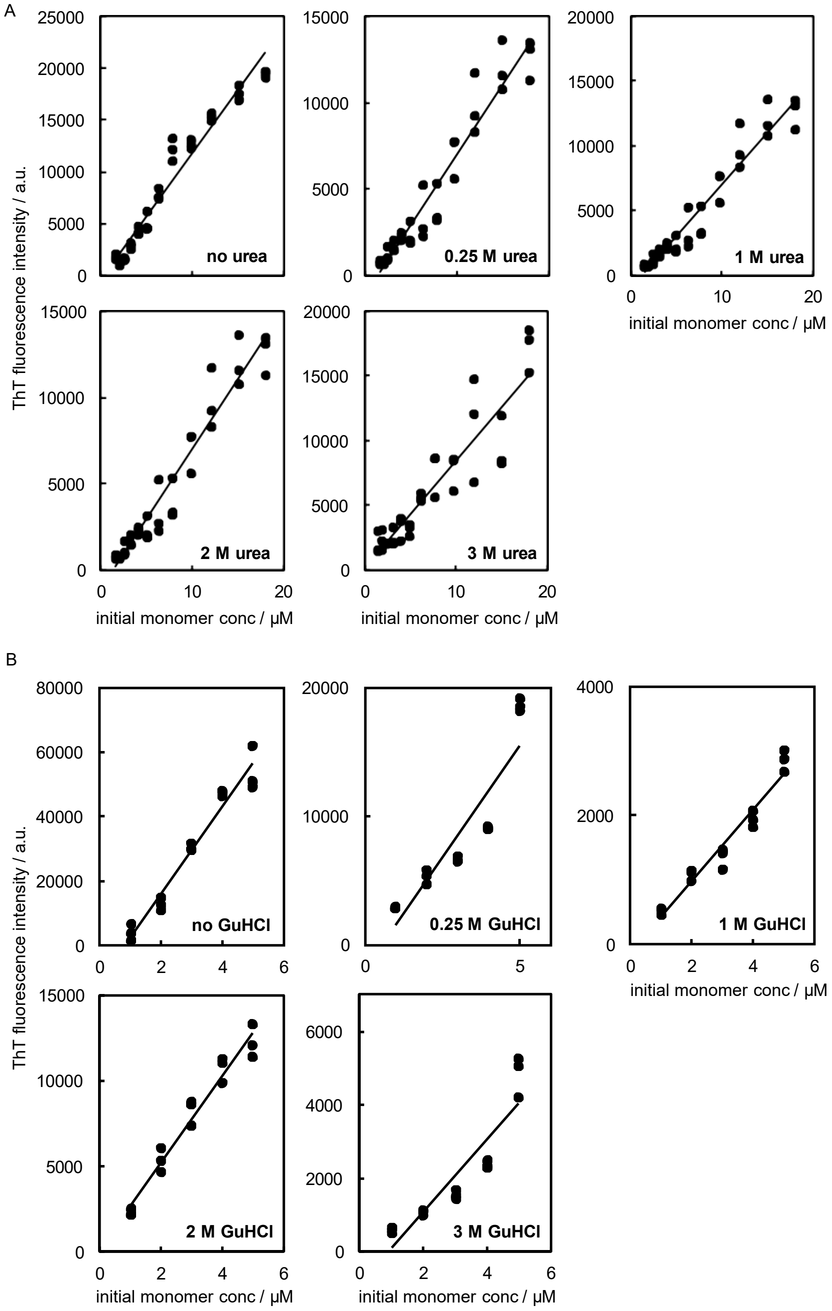

Figure S5. Scaling of ThT fluorescence with total aggregate mass. The average fluorescence intensity is plotted against the total monomer concentrations in urea (A) and in GuHCl (B). The fluorescence intensity scales linearly with the amount of monomer at all denaturant concentrations.

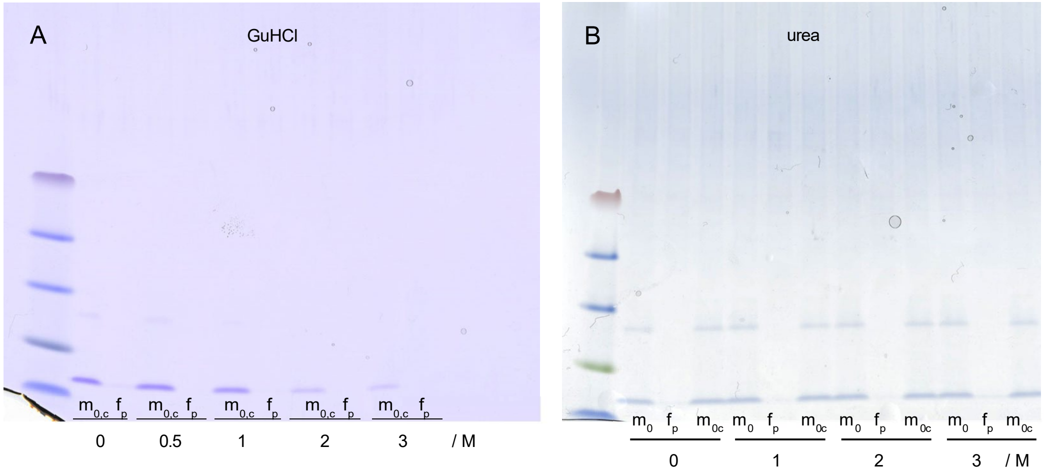

Figure S6. SDS-PAGE analysis of fibrillar mass. Aβ42 aggregation in GuHCl (A) and urea (B) was followed by removal of fibrils by centrifugation and the supernatants were analysed by SDS PAGE on a 4-20% Tris/tricine gel. Identical monomeric samples, $m_{0,c}$, were applied followed by samples obtained from the plateau phase (f_p) of the aggregation reaction. No monomers could be detected after aggregation implying that most of the monomers are consumed and that the decrease in ThT signal is not due to a shift in equilibrium between monomers and fibrils. Monomeric samples not subjected to centrifugation (m_0) was also applied to gel B. Sodium dodecyl sulphate (SDS) was excluded from the solution buffer for the Aβ42 samples aggregating in GuHCl due to precipitation.

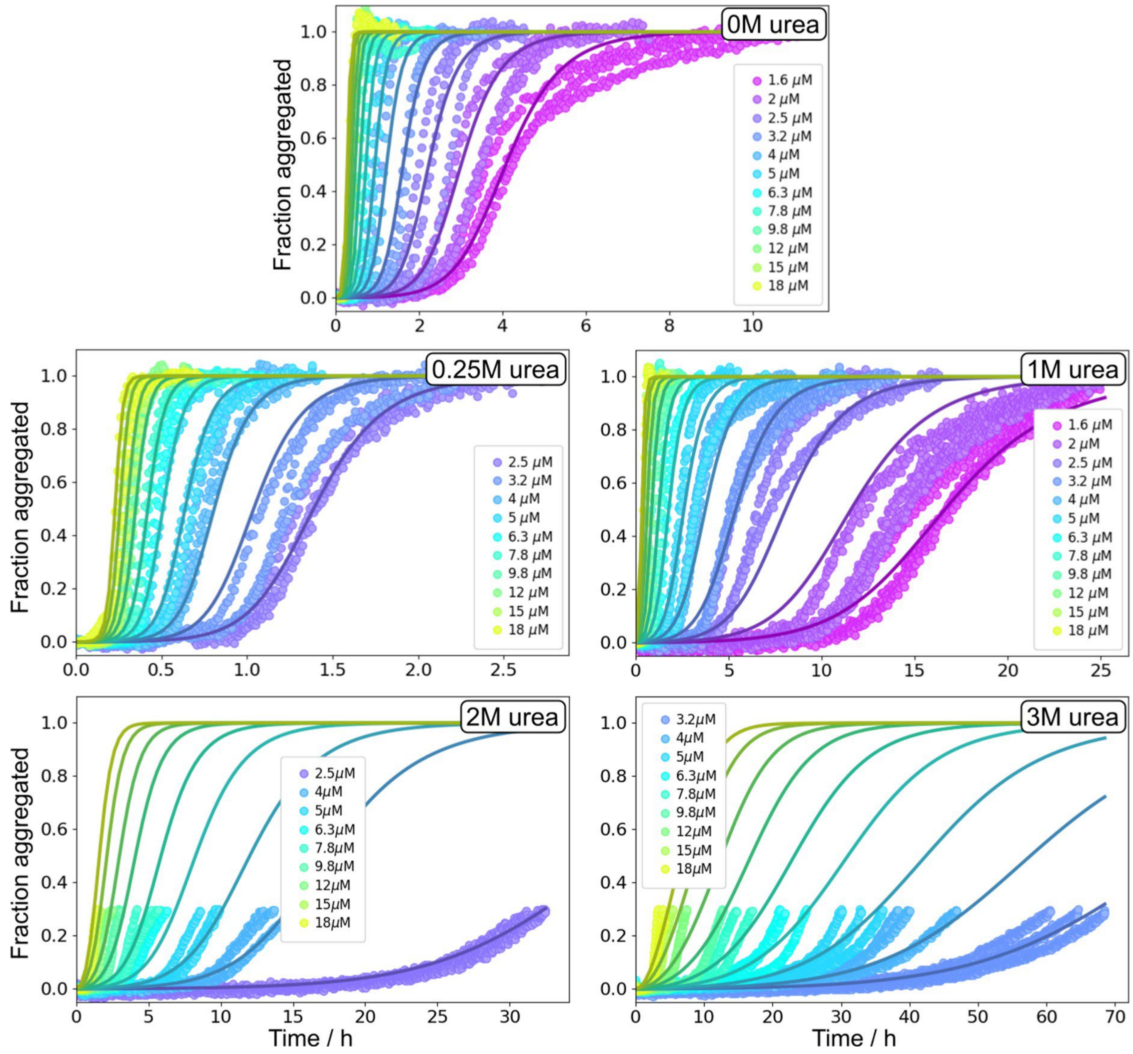

Figure S7. Global fitting of additional data set of Ab42 aggregation in 0 M urea, 0.25 M urea, 1 M urea, 2 M urea and 3 M urea in 20 mM sodium phosphate, 0.2 mM EDTA, 0.02% sodium azide, pH 8.0.

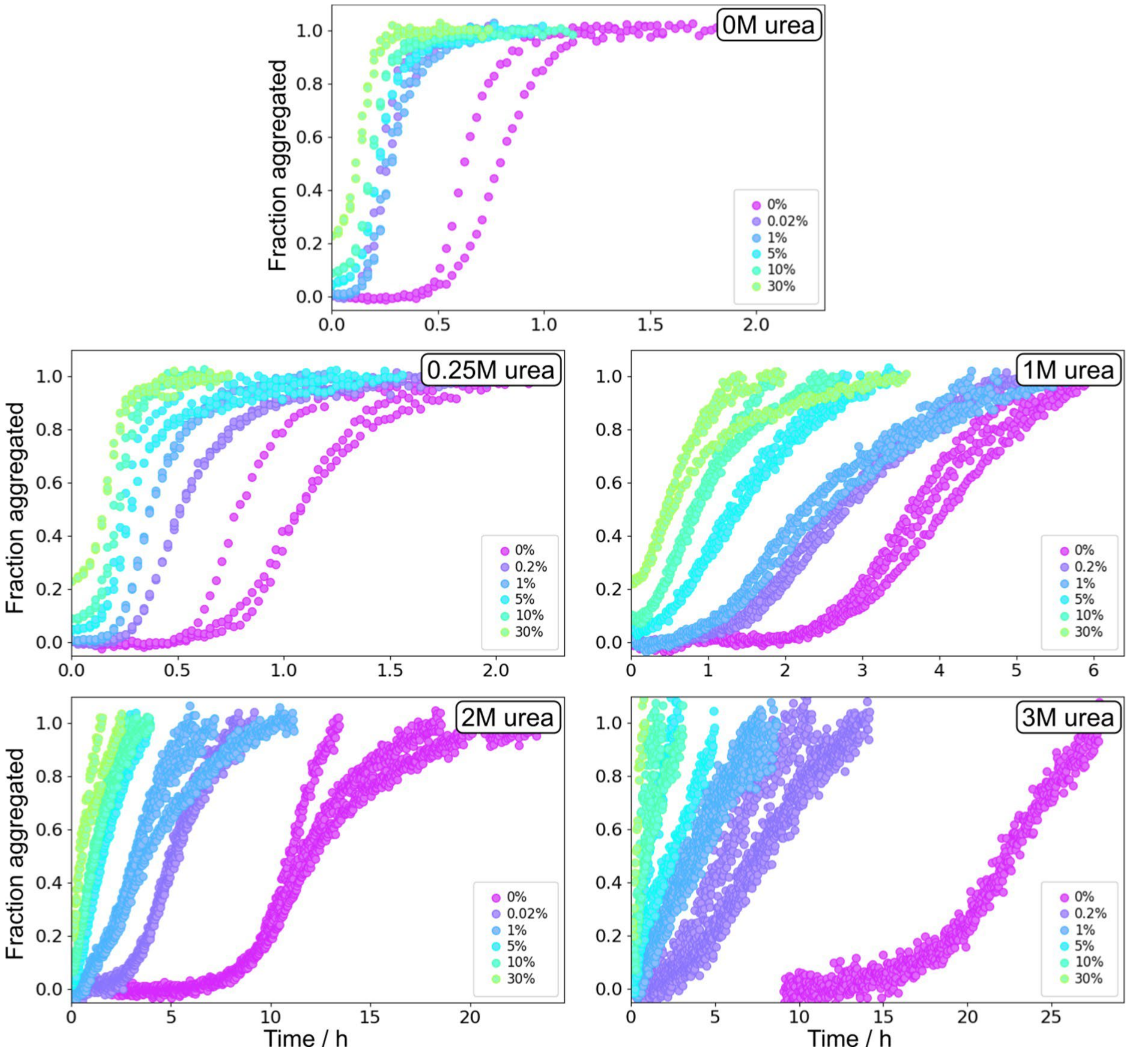

Figure S8. Additional seeding experiments in urea. Normalized ThT fluorescence as a function of time for 3 μM Aβ42 in buffer with no urea, 1 M urea, 2 M urea and 3 M urea in 20 mM sodium phosphate, 0.2 mM EDTA, 0.02% sodium azide, pH 8.0. Seeds, prepared at the corresponding urea concentration, were added at time zero at concentrations of 0.2%, 1%, 5%, 10% and 30% in monomer equivalents.

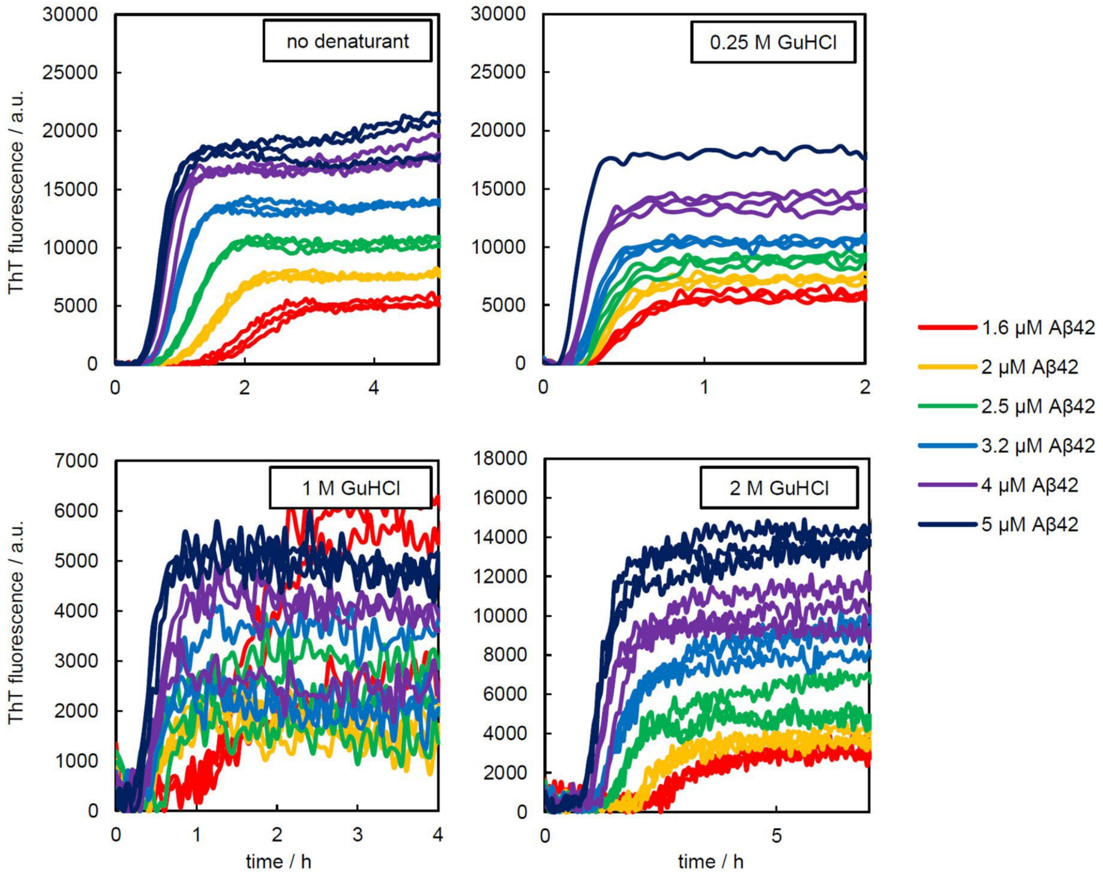

Figure S9. Concentration dependent aggregation of Aβ42 in GuHCl. The aggregation process of Aβ42 was monitored by ThT fluorescence intensity as a function of time without denaturant , in 0.25 M , 1 M and in 2 M GuHCl. Different gains were set on the plate reader in presence and absence of GuHCl since the signal was quenched by addition of GuHCl.

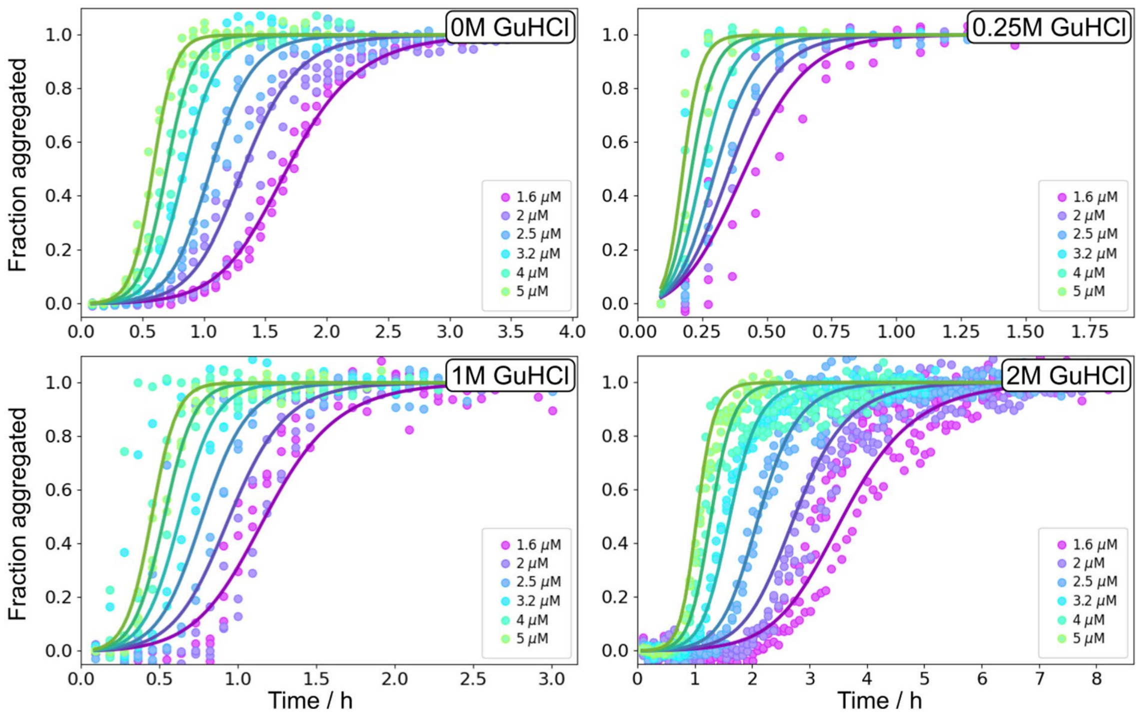

Figure S10. Global fitting of additional data set of Aβ42 aggregation in 0 M GuHCl, 0.25 M GuHCl, 1 M GuHCl and 2 M GuHCl in 20 mM sodium phosphate, 0.2 mM EDTA, 0.02% sodium azide, pH 8.0.
